# Individualized Corneal Patching for Treatment of Corneal Trauma Combined with Tissue Defects

**DOI:** 10.1155/2020/8437479

**Published:** 2020-11-23

**Authors:** Ting Zhang, Yanni Jia, Suxia Li, Weiyun Shi

**Affiliations:** ^1^Eye Hospital of Shandong First Medical University, Jinan, China; ^2^State Key Laboratory Cultivation Base, Shandong Provincial Key Laboratory of Ophthalmology, Shandong Eye Institute, Shandong First Medical University & Shandong Academy of Medical Sciences, Qingdao, China

## Abstract

**Aim:**

To evaluate the efficacy of individualized corneal patching using a minimal graft for corneal trauma combined with tissue defects.

**Methods:**

Fifteen eyes (15 patients) were enrolled in this study, including 8 eyes with corneal perforation induced by removal of metal foreign bodies, 5 eyes with corneal laceration resulting from metal trauma, and 2 eyes with pencil injuries to the cornea. The size, shape, and depth of the tissue defects were assessed. For corneal perforation or irregular tissue defects, if the diameter or length was ≥3.0 mm, traditional penetrating keratoplasty (PK) or lamellar keratoplasty (LK) was adopted; if the diameter or length was ＜3.0 mm, a conical or irregular patch consistent with the defects was used. The visual acuity, corneal status, and postoperative complications were observed during the follow-up.

**Results:**

The diameter of corneal perforations was 1.0 mm in 2 eyes, 1.5 mm in 1 eye, 2.0 mm in 4 eyes, and 3.5 mm in 1 eye. During their PK procedures, a conical corneal graft was used in 7 eyes, while a traditional cylindrical graft was used in 1 eye. The other 7 eyes had corneal trauma combined with irregular tissue defects, which were full-thickness corneal defects in 5 eyes and lamellar defects in 2 eyes, all less than 3.0 mm in length. Thus, five eyes received PK, and 2 eyes received LK using an irregular wedge-shaped patch. The visual acuity increased greatly postoperatively, with mild corneal astigmatism. None of the patients developed immune rejection.

**Conclusion:**

Individualized corneal patching with a minimal graft can save corneal materials, relieve corneal scars, gain a good visual prognosis, and avoid immune rejection in the treatment of corneal trauma combined with tissue defects.

## 1. Introduction

Corneal laceration and corneal perforation are common ocular traumas with potentially devastating sequelae, including corneal scarring, astigmatism, and endophthalmitis [1]. Generally, the risk of blindness due to endophthalmitis is quite low, but the visual loss caused by high astigmatism related to corneal scars is frequently encountered. Irregularly shaped corneal lacerations are usually associated with poorer prognoses after surgery than regularly shaped ones, especially those with partial corneal defects or corneal perforation, which often put ophthalmic doctors in a dilemma. In the situation, either an improper juxtaposition of the wound edges or an influence on the corneal curvity might cause severe corneal scarring and high irregular astigmatism.

Depending on the features of corneal injuries, multiple approaches can be taken to repair the cornea. In cases of small or self-sealed corneal perforation or corneal laceration, corneal bandage lenses can be used to promote epithelial healing [1], but they may fail to prevent aqueous humor leakage when the lacerations or perforations are large [2]. Suturing is effective against lacerations, but if partial corneal defects coexist, tight stitching of the wound is likely to result in decreased vision due to high irregular astigmatism [3]. Moreover, utilities of amniotic membrane, cyanoacrylate, or fibrinogen adhesives as a packing material have been reported [4–6]. Nevertheless, they also tend to be useless in large corneal perforations.

For corneal injuries accompanied by damaged corneal tissue, repairing the lesion with a same-sized corneal patch may help to reestablish the corneal structure and curvature. Herein, we investigated the efficacy of individualized corneal patching with a minimal graft for the treatment of corneal injuries with tissue loss.

## 2. Materials and Methods

### 2.1. Patients

This study was approved by the Institutional Review Board of Eye Hospital of Shandong First Medical University. Written informed consent was obtained from all participants. Medical records of patients who were treated by individualized corneal patching for corneal injuries combined with tissue defects at our hospital between April 2015 and March 2018 were retrospectively reviewed. There existed no concurrent corneal infection, traumatic cataract, intraocular foreign body, vitreous hemorrhage, or retinopathy.

Fifteen patients (15 eyes) were included in the study. Eight patients had corneal perforation induced by removal of full-thickness corneal metal foreign body, and 3 of them had received conjunctival flap covering, including 2 with tight stitching of the cornea. Five patients had corneal laceration induced by metal trauma and were associated with improper saturation or wound leakage. Two patients presented with corneal laceration induced by pencil pricking. Fourteen of the 15 patients had been treated at local hospitals; with one eye had received saturation three times, six eyes had received surgery twice, and 7 eyes had received surgery once. However, the cornea remained distortured or unhealed.

### 2.2. Corneal Trauma Evaluation

Before surgery, the size, morphology, and depth of corneal tissue defects were accessed using slit-lamp biomicroscopy and the anterior segment optical coherence tomography (AS-OCT, Optovue, RTVue100-2, Fremont, CA, USA), then different ways of corneal patching were adopted accordingly. Moreover, if the wound was covered with a conjunctival flap or the patient was a child, a detailed assessment of the wound was performed intraoperatively.

### 2.3. Surgical Principle

A comprehensive assessment of the etiology and the wound architecture would be helpful to design an individualized surgical procedure. For instance, corneal perforations induced by removal of full-thickness corneal foreign body often had conical tissue defects, with the anterior surface larger than the posterior surface, for which a conical corneal patch was usually needed. Irregularly polygonal corneal tissue defects resulting from trauma were commonly more severe at the anterior surface than the posterior surface due to traumatic shearing forces and thus were also featured with larger anterior surface tissue defects, for which an irregular wedge-shaped graft was demanded.

For corneal perforation or irregular polygonal corneal tissue defects, if the diameter of perforation or the length of tissue defects was ≥3.0 mm, traditional penetrating keratoplasty (PK) or lamellar keratoplasty (LK) with a cylindrical graft was adopted. If the diameter of perforation or the length of tissue defects was ＜3.0 mm, a conical or an irregular wedge-shaped patch consistent with the shape and depth of the tissue defects was used.

### 2.4. Corneal Patch Preparation

When preparing a conical corneal patch, the trephination of the donor cornea was performed using a trephine from the endothelial side in the diameter of the anterior surface of the tissue defects and with a depth of 2/3 of the corneal thickness. With the endothelial side up, the posterior surface of the patch was then carefully excised into a cone, after which the complete corneal patch was cut off.

When making an irregular wedge-shaped patch, the auxiliary arm of a caliper was used to mark on the donor cornea according to the specific shape of the tissue defects in the corneal anterior surface. The corneal patch was cut vertically following the mark before its posterior surface was trimmed carefully to fit the wound. When the patch was for lamellar grafting, a part of the posterior stroma was excised in accordance with the tissue defects depth before the trimming of the posterior surface. All the donor corneas (glycerol cryopreserved) were obtained from the Eye Bank of Eye Hospital of Shandong First Medical University.

### 2.5. Surgical Technique

All surgeries were performed under retrobulbar or general anesthesia by the same surgeon (WS). In the patients with previous surgery of corneal laceration suturing, conjunctival flap covering, or amniotic membrane packing, the sutures or amniotic membrane was removed, and the conjunctival flap was retracted to release the corneal tension. Then, the characteristic of the corneal wound including the shape, size, and depth of tissue defects was further evaluated.

**Figure 1:**
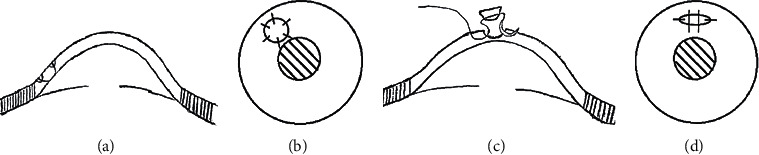
Corneal patching with a conical/irregular wedge-shaped graft. (a-b) Penetrating corneal patching with a conical graft. The graft is sutured onto the host bed with interrupted sutures. (c-d) Lamellar corneal patching using an irregular wedge-shaped graft. The graft width is ＜1.5 mm, and the suture starts from the host cornea to the graft and finally through the host cornea on the opposite side. This continuous suture method is also applicable for penetrating corneal patching.

For corneal perforation or full-thickness corneal laceration, the necrotic tissue attaching to the injury was removed, after which penetrating corneal patching was performed. Firstly, a 1.0 mm limbal paracentesis incision was made using a diamond knife, from which the miotic agent and sodium hyaluronate (10 mg/mL Healon) were sequentially injected into the anterior chamber. Meanwhile, the iris incarcerated in the corneal wound was returned into the anterior chamber with the injection of Healon. Next, a trephine or caliper was used to mark the margin of the tissue defects on the injured cornea, and the size of the defects was confirmed. Then, the trephine or caliper was pressed onto the donor cornea to make a boundary of the patch shaped like the tissue defects, and the individualized conical patch or irregular wedge-shaped patch was made as previously described before it was fixed to the recipient bed with interrupted 10–0/11–0 monofilament nylon sutures (Figure 1). After the anterior chamber was filled with a balanced salt solution, and the suture was rotated into the stroma, a soft contact lens was placed on the cornea. For patients requiring a partial thickness corneal patching, the necrotic tissue attaching to the injury was removed, after which the patch was fixed to the recipient bed with interrupted 10–0/11–0 monofilament nylon sutures.

With respect to the saturation, if the diameter of the conical patch or the width of the irregular wedge-shaped patch was ≥1.5 mm, junction of the graft and host cornea was sutured using interrupted sutures. If the diameter or the width of the patch was <1.5 mm, continuous suturing following a host-graft-host path was performed (Figure 1).

### 2.6. Postoperative Treatment

Postoperatively, tobramycin and dexamethasone eye ointment (Alcon, Puurs, Belgium) was used 2–4 times daily for 1–2 weeks and thereafter replaced with 0.1% fluorometholone eye drops (Santen, Osaka, Japan) 4 times daily for 1-2 months, followed by 0.02% fluorometholone eye drops 2–4 times daily (Santen, Osaka, Japan). If necessary, 1% cyclosporine eye drops (Huabei Pharmaceutical Co., Shijiazhuang, China) were administered once or twice per day. The medication was adjusted with the alleviation of inflammation.

### 2.7. Postoperative Evaluation

All patients underwent examinations daily for the first week, weekly for 4 weeks, and monthly thereafter. The uncorrected visual acuity (UCVA), best corrected visual acuity (BCVA), spherical equivalent, astigmatism, and intraocular pressure (IOP) were recorded. The graft status and corneal thickness were inspected by AS-OCT. Postoperative complications and immune rejection were observed during the follow-up.

### 2.8. Statistical Analysis

The data were analyzed using SPSS® 11.5 software. The UCVA and BCVA before and after surgery were compared with the paired *t*-test. A *P* value of <0.05 was considered to be statistically significant.

## 3. Results

### 3.1. General Information

Among the 15 patients, 13 were male, and 2 were female, with an age range of 4–57 years (mean ± standard deviation, 35.9 ± 15.7). All the corneal traumas were combined with tissue defects which had larger anterior surfaces than the posterior surfaces.

In the case series, 8 eyes had corneal perforation induced by removal of metal foreign body, 5 eyes had corneal laceration resulting from metal trauma, and 2 eyes had pencil injury to the paracentral cornea. Among the 8 eyes with corneal perforation, the diameter of perforation was 1.0 mm in 2 eyes, 1.5 mm in 1 eye, 2.0 mm in 4 eyes, and 3.5 mm in 1 eye; 7 eyes received PK with a conical graft, and 1 eye, in which the diameter of the perforation was 3.5 mm, received PK with a cylindrical graft.

The other 7 eyes had corneal trauma combined with irregular tissue defects of less than 3.0 mm in length. Five eyes with irregular full-thickness corneal tissue defects were treated by PK, whereas 2 eyes with irregular lamellar corneal tissue defects received LK using an irregular wedge-shaped patch (Table 1).

### 3.2. Clinical Examination

**Figure 2:**
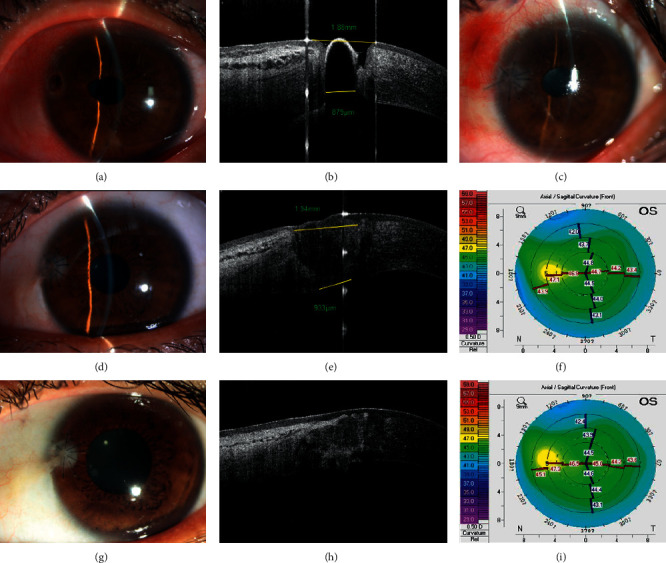
Penetrating corneal patching with a conical graft for corneal perforation. (a) Corneal perforation at 10 o'clock, with the UCVA being 0.22 logMAR. (b) AS-OCT indicates that the tissue defects' diameter of the anterior and posterior surfaces is 1.88 mm and 897 *μ*m, respectively. (c) A photograph at 1 week after surgery. (d–f) Examination at 12 months after surgery. (d) The graft opacity is slightly increased, and the UCVA is 0.15 logMAR. (e) AS-OCT displays the profile of the corneal patch, with the diameter in the anterior surface being 1.94 mm and the posterior surface being 933 *μ*m. (f) The curvature of the front cornea is 44.7/45.7. (g–i) Examination at 24 months after surgery. (g) The graft opacity is increased, and the UCVA is 0.097 logMAR. (h) AS-OCT displays the profile of the corneal patch. (i) The curvature of the front cornea is 44.5/45.6.

**Figure 3:**
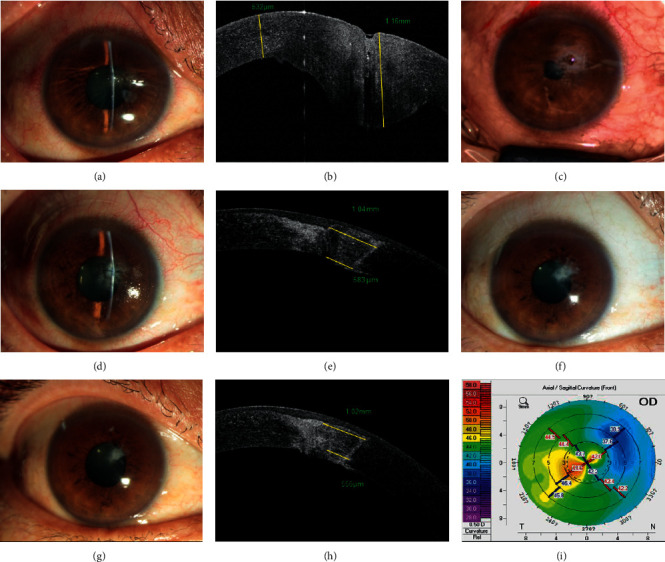
Penetrating corneal patching using an irregular wedge-shaped graft for cornea laceration combined with tissue defects. (a) Radial ruffles form in the central cornea because of tight stitching, with the UCVA being 1.0 logMAR. (b) AS-OCT indicates distortion of the corneal contour. (c) Morphology of tissue defects after suture removal. (d) A photograph at 1 week after surgery. (e–f) Examination at 8 months after surgery. (e) AS-OCT shows that the width of the anterior surface of the patch is 1.04 mm, and that of the posterior surface is 583 *μ*m. Scar forms in the injured cornea at the temporal side of the patch. (f) The graft opacity is slightly increased, and the UCVA is 0.30 logMAR. (g–i) Examination at 24 months after surgery. (g) The graft opacity is slightly decreased, with the UCVA being 0.15 logMAR. (h) AS-OCT shows the profile of the corneal patch. (i) The curvature of the front cornea is 43.0/45.9.

**Figure 4:**
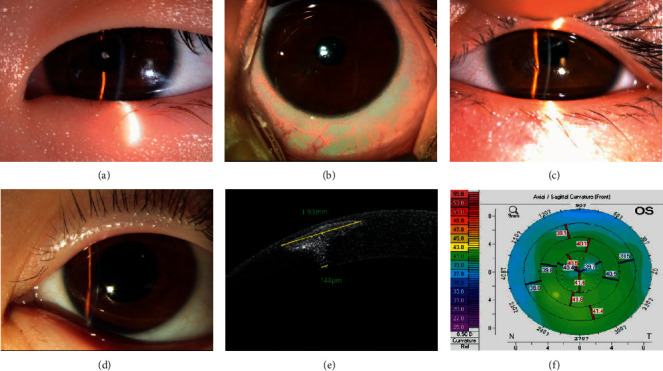
Penetrating corneal patching with an irregular wedge-shaped patch for pencil pricking. (a) The sutured cornea is packed with the amniotic membrane in the wound, with secretions accumulating on the central cornea, and the UCVA is 1.0 log MAR. (b) Morphology of tissue defects after removing the sutures and amniotic membrane. (c) A photograph at 1 week after surgery. (d–f) Examination at 4 years after surgery. (d) The graft opacity is decreased, and the UCVA is 0.1 logMAR. (e) AS-OCT shows that the longitudinal of the anterior surface of the patch is 1.93 mm, while the width of the posterior surface is 144 *μ*m. (f) The curvature of the front cornea is 40.0/41.0.

**Figure 5:**
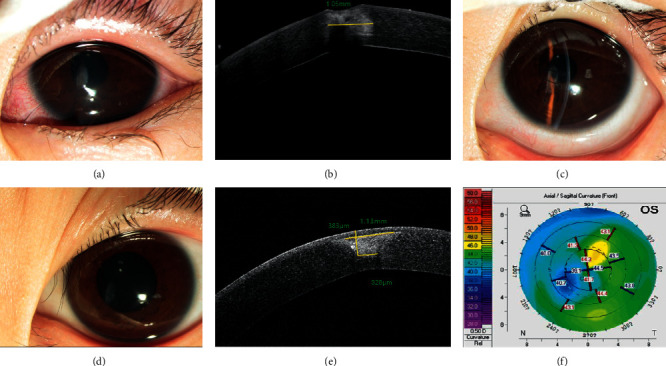
Lamellar corneal patching with an irregular wedge-shaped graft for pencil pricking. (a) The lamellar laceration is in the central cornea with stromal edema, and the UCVA is 1.0 logMAR. (b) AS-OCT shows inflammatory infiltration in a range of 1.05 mm. (c) Photograph at 1 week after surgery. (d–f) Examination at 4 years after surgery. (d) The graft opacity is decreased, and the UCVA is 0.22 logMAR. (e) AS-OCT shows that the width of the anterior surface of the patch is 1.13 mm, and that of the posterior surface is 328 *μ*m. The anteroposterior diameter of the patch is 383 *μ*m. (f) The curvature of the front cornea is 42.3/43.7.

**Figure 6:**
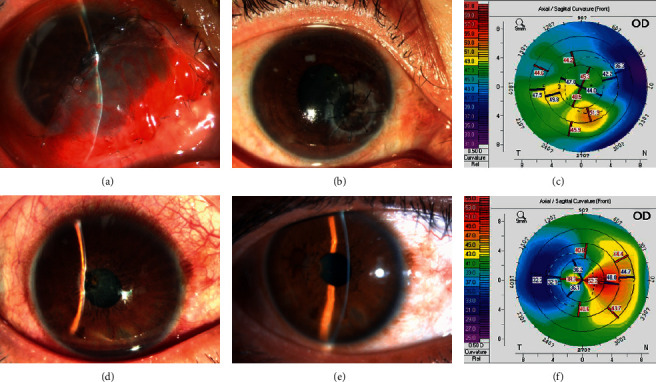
Penetrating keratoplasty and corneal patching for corneal perforation. (a–c) Penetrating keratoplasty with a cylindrical graft of 3.5 mm in diameter. (a) Half of the cornea is covered by a conjunctival flap, and the anterior chamber disappears, with UCVA being hand movement. (b–c) Eight months after surgery, corneal edema is alleviated greatly, the UCVA is 1.0 logMAR, and the curvature of the front cornea is 47.3/47.7. (d–f) Penetrating corneal patching with a conical graft. (d) Corneal perforation at the central cornea, with no anterior chamber, and the UCVA being 1.22 logMAR. (e–f) 12 months after surgery, the corneal patch is transparent, the UCVA is 0.40 logMAR, and the curvature of the front cornea is 38.7/44.5.

Of the 15 eyes, two eyes had a lamellar corneal laceration, two eyes had a full-thickness corneal injury which was stitched tightly, and the other 11 eyes had aqueous humor leakage and presented with hypotony before surgery. Following surgery, the anterior chamber depths recovered, the IOP returned to normal levels, and the Seidel tests were negative. The eye hyperemias decreased gradually. The corneal edemas subsided within 2 weeks. Elevated IOP (28 mmHg) occurred in 1 eye and was controlled by medication within 2 days. The mean corneal thickness of the central patching area was 598 *μ*m with a range of 509–750 *μ*m. During the follow-up, slight graft opacity was found in two cases but rarely affected the visual acuity. A good apposition at the junction of the graft and host cornea ensured the healing of the wound. Visual acuity was improved greatly in all eyes, with mild corneal astigmatism (Figures 2–6).

### 3.3. Recovery of Visual Acuity 

Before and after surgery, the average UCVA was 1.17 logMAR and 0.51 logMAR, respectively (*t* = 4.932, *P* < 0.001, Table 2), and the average BCVA was 0.96 logMAR and 0.29 logMAR, respectively (*t* = 4.581, *P* < 0.001, Table 2). The difference in UCVA/BCVA pre- and postoperatively were both statistically significant. The mean value of keratometric astigmatism was 2.38 diopters after surgery, with a range of 0.25–5.80 diopters.

### 3.4. Complications

All the grafts were minimal, so most patients did not need cyclosporine eye drops, and 0.02% fluorometholone eye drops were administered for about one year and a half. After the sutures were removed, only artificial tear eye drops were used if necessary. There was no secondary infectious keratitis throughout the mean follow-up of 17.7 months. Corneal graft epithelial defects, which may be attributed to the graft protuberance at the graft-host junction, were observed in 2 eyes and cured with suture once and twice, respectively, to attain corneal epithelialization. Corneal graft opacity and neovascularization at the graft-host junction were detected in 2 eyes and were alleviated gradually with the removal of sutures and the use of corticosteroid eye drops.

## 4. Discussion

As corneal structure and corneal curvature play important roles in the eye's visual function, irregular corneal wound is difficult to repair, especially when the wound is combined with corneal tissue defects or corneal perforation. For these eyes, conventional suturing may not solve the wound leakage, while tight suturing usually results in unacceptable distortion of the corneal contour.

It has been shown in previous studies that tissue adhesives were effective in closing small corneal perforation and stabilizing corneal integrity in a short-term time, but almost half of the patients required surgical intervention within 90 days. Moreover, when the corneal perforation was large, tissue adhesive application had a high rate of failure [7–9]. For the management of corneal laceration, tissue adhesives can treat partial thickness corneal wounds and full-thickness linear or stellate corneal wounds as a viable alternative to conventional sutures [10–12]. However, their therapeutic effects on corneal laceration combined with tissue defects have never been reported.

Furthermore, treating corneal perforation with a conjunctival flap covering remains controversial for the flap may fail to seal the wound and would affect the visual acuity and appearance. Tight stitching of the wound in eyes with corneal perforation to restore the ocular integrity might cause radial ruffle in cornea and result in high irregular astigmatism. Although there are various ways to correct astigmatism after surgery, such as rigid gas permeable contact lenses [13, 14], it is imperative to avoid the formation of large astigmatism during surgery rather than to correct it postoperatively.

Individualized corneal patching with a minimal graft introduced in the current study can effectively treat corneal perforation or corneal injury combined with tissue defects in varied sizes and shapes. In fact, trauma rarely results in large corneal perforation or tissue defects. We treated the patients using corneal patches of approximately the same size as the tissue defects, which was minimally 1.0 mm in diameter. In this condition, it would be difficult to apply radial sutures in keratoplasty because the graft was too small. Therefore, we adopted a continuous suturing following a host-graft-host path. In the case series, all the corneas were repaired with minimal grafts in a conical, cylindrical, or irregular wedge shape. This individualized surgery significantly improved the visual prognosis and saved the donor corneal tissue.

To successfully fulfill the individually designed surgery, AS-OCT, a nontouch technique, could be used to demonstrate the structure of corneal wound preoperatively and analyze the profile at the graft-host junction postoperatively [15, 16]. The precise apposition at the graft-host junction is an important target of keratoplasty and a prognostic factor affecting visual outcomes [17].

Experience of the surgeons plays an important role in the prognosis of complex corneal traumas, since the trephination of the recipient bed and donor material, excessive or uneven suture tension, graft-host misalignment, and unsatisfactory wound healing all might influence the visual outcome [18–20]. In this study, each graft-host interface was scanned, and all patients achieved a well-apposed graft-host junction.

Individualized corneal patching with a minimal graft could achieve anatomical and functional success in treating corneal trauma combined with tissue defects. And the procedure is suitable for both central and paracentral corneal injuries. Compared to PK or LK using a large graft, corneal patching with a minimal graft can reduce the occurrence of immune rejection. Using a conical or wedge-shaped graft also maximally preserves the recipient corneal endothelium, which simultaneously benefits the healing of the cornea. Furthermore, in the regions with a paucity of donor materials, individualized corneal patching helps to save donor corneas and make more people gain the opportunities of vision restoration. Although the outcomes of the surgery were good in current study, a randomized, controlled study with larger sample size is needed for further investigation.

## 5. Conclusion

Repairing the perforation and corneal tissue defects with the same-sized donor graft as a patch could minimize corneal scarring and maximally restore the anatomical structure of the eye globe, so as to guarantee a better prognosis. This technique is likely to be useful in preserving long-term corneal stability and good visual function in the treatment of corneal perforation and corneal injury combined with tissue defects.

## Figures and Tables

**Table 1:** Summary of clinical data.

Patient	Age (yrs)/Sex	Cause	Indication for surgery	Previous surgery/frequency	Graft feature/surgery
1	32/M	FTFB	Corneal perforation	Suture and conjunctival flap covering/1	Conical patch/PK
2	49/M	FTFB	Corneal perforation	FTFB removal, FTFB removal and conjunctival flap covering/2	Conical patch/PK
3	28/M	FTFB	Corneal perforation	FTFB removals/2	Conical patch/PK
4	28/M	FTFB	Corneal perforation	FTFB removals/2	Conical patch/PK
5	44/M	FTFB	Corneal perforation	FTFB removals/2	Conical patch/PK
6	41/M	FTFB	Corneal perforation	FTFB removal/1	Conical patch/PK
7	57/M	FTFB	Corneal perforation	FTFB removals/2	Conical patch/PK
8	41/F	FTFB	Corneal perforation	Suture, suture and conjunctival flap covering/2	Cylindrical patch/PK
9	38/M	Still wire puncture	Wound leakage	Suture/1	Irregular wedge-shaped patch/PK
10	53/M	Iron sheet injury	Wound leakage	Suture/1	Irregular wedge-shaped patch/PK
11	26/M	Aluminum wire puncture	Improper saturation	Suture/1	Irregular wedge-shaped patch/PK
12	41/M	Iron nail hurt	Improper saturation	Sutures/3	Irregular wedge-shaped patch/PK
13	49/M	Steel bar hurt	Improper saturation	Suture/1	Irregular wedge-shaped patch/LK
14	5/F	Pencil pricking	Full-thickness corneal laceration	Suture and AM packing/1	Irregular wedge-shaped patch/PK
15	4/M	Pencil pricking	Lamellar corneal laceration	None	Irregular wedge-shaped patch/LK

FTFB, full-thickness foreign body; PK, penetrating keratoplasty; LK, lamellar keratoplasty; AM, amniotic membrane.

**Table 2:** The difference in visual acuity (log MAR) before and after surgery.

Data	Mean	SD	*N*	*t*	*P* value^a^	95% CI
Difference of UCVA	0.66	0.52	15	4.932	0.0002	0.37–0.95
Difference of BCVA	0.67	0.57	15	4.581	0.0004	0.38–0.99

UCVA: uncorrected visual acuity, BCVA: best corrected visual acuity, SD: standard deviation, *N*: number of eyes, CI: confidence interval. ^a^Paired *t*-test.

## Data Availability

The data used to support the findings of this study are available from the corresponding author upon request.
